# Contribution of Anemia to Multidimensional Indices for Predicting Mortality in Hospitalized Patients With Chronic Obstructive Pulmonary Disease (COPD)

**DOI:** 10.7759/cureus.72126

**Published:** 2024-10-22

**Authors:** E Garcia-Pachon, Isabel Padilla-Navas

**Affiliations:** 1 Section of Respiratory Medicine, Hospital General Universitario de Elche, Elche, ESP

**Keywords:** anemia, copd, mortality, prediction, prognosis, survival

## Abstract

Background: Severe exacerbations are a significant predictor of poor prognosis and mortality in patients with chronic obstructive pulmonary disease (COPD). Multidimensional indices, such as the BODE (BMI, airflow obstruction, dyspnea, and exercise capacity) and ADO indices (age, dyspnea severity, and airflow obstruction), outperform single-variable assessments in predicting survival. However, anemia, a strong predictor of mortality in both the general population and COPD patients, has not been included in the prediction indices. This study aimed to evaluate whether including anemia as a variable enhances the predictive accuracy of these indices for both short-term and long-term mortality in COPD patients.

Methods: Data from patients who were consecutively admitted for acute exacerbation of COPD were recorded with a minimum follow-up of three years. Patients were divided into two groups: anemic (Hb <12 g/dL in women and Hb <13 g/dL in men) or non-anemic. Modified versions of the BODEx (BMI, airflow obstruction, dyspnea, and exacerbation) and ADO indices that included anemia, termed BODEx-A3 and ADO-A3, were created by adding three points to the original values.

Results: A total of 141 patients were included. Twenty-one (15%) died during the first year after admission, and 48 (34%) died during the three-year follow-up period. The area under the receiver operating characteristic curve (AUC) for predicting one-year mortality was slightly higher with the BODEx-A3 compared to the BODEx (0.83 vs. 0.78) and with the ADO-A3 compared to the ADO (0.81 vs. 0.78). For three-year mortality, the predictive power of the BODEx-A3 (AUC 0.77 vs. 0.67 for BODEx, p<0.001) and ADO-A3 indices (AUC 0.82 vs. 0.77 for ADO, p=0.038) was significantly greater compared to their original versions.

Conclusion: We have defined novel multidimensional indices (BODEx-A3 and ADO-A3) for predicting short- and long-term mortality in patients hospitalized with COPD exacerbation. These indices were derived by adding three points to the standard BODEx and ADO scores in anemic patients. Our findings demonstrate that incorporating anemia into the best existing COPD mortality predictor indices significantly improves their predictive capacity.

## Introduction

Chronic obstructive pulmonary disease (COPD) is the third most common cause of death worldwide [1]. Severe exacerbations of COPD requiring hospitalization are a well-established predictor of worse prognosis, including mortality [2,3]. To provide adequate care to patients who have been admitted for exacerbation of COPD, it may be useful to have resources to predict the risk of mortality. A number of clinically significant factors predicting mortality after admission for COPD exacerbation have been identified [4]. However, it appears that the suggested criteria usually proposed for initiating palliative care do not offer sufficient reliability [5].

It has been demonstrated that multidimensional indices are better predictors of survival than either variable separately. Several indices that include clinical data have been evaluated, and the BODE-derived indices and the ADO index have been reported as the best simple multicomponent indices to predict survival [6-8].

The original BODE index incorporates BMI (B), airflow obstruction (O), dyspnea (D), as measured by the modified Medical Research Council (mMRC) scale, and exercise capacity (E), as measured by the six-minute walk distance (6MWD). However, 6MWD may not always be available, and a modification replacing this variable with exacerbations has been successfully proposed [9], the BODEx index (BMI, airflow obstruction, dyspnea, and exacerbation). The multidimensional ADO index includes an assessment of age (A), dyspnea severity (D), and O (obstruction - FEV1) [10]. Both the BODEx index and the ADO index are of considerable practical interest because of their simplicity and ease of calculation.

Anemia is a well-known risk factor for mortality in the general population irrespective of age, gender, or cardiovascular disease history [11] and specifically in COPD patients requiring hospitalization [12]. However, this parameter has not been evaluated in these multidimensional indices.

The objective of this study was to assess whether incorporating anemia as a variable enhances the prediction of mortality in the most widely used multidimensional indices. We aimed to evaluate this potential improvement in both short-term mortality prediction (within one year) and long-term mortality prediction (within three years).

## Materials and methods

Data from patients who were consecutively admitted to the pulmonology department between July 2019 and May 2021 for acute exacerbations of COPD were recorded. The follow-up deadline was May 31, 2024, ensuring a minimum of three years of follow-up from the date of each patient’s first admission at the time of data analysis.

The diagnosis of COPD was based on age ≥40 years, a current or past smoking history (≥10 pack-years), and post-bronchodilator FEV1/FVC <70%, according to standard diagnostic criteria. When patients required more than one admission during the inclusion period, only the data from the first admission were recorded. Patients with concurrent cancer or interstitial lung disease were not included. Those patients who could not be followed up during the established period were excluded.

At the time of admission, the following variables were documented: age, gender, BMI, calculated as weight in kilograms divided by the square of height in meters, smoking history, spirometric measurements, and the number of hospitalizations and emergency department visits in the previous year. Baseline dyspnea, under stable conditions, was evaluated using the mMRC dyspnea scale. The date of death was obtained from electronic medical records.

Hematological parameters were assessed from the initial blood sample collected during routine clinical testing. Patients were divided into two groups based on hemoglobin (Hb) levels, classified as either anemic or non-anemic. Anemia was defined according to the criteria set by the World Health Organization [13]: Hb <12 g/dL for women and Hb <13 g/dL for men.

The BODEx and ADO indices were calculated for each patient based on standard criteria [9,10]. The new versions of these multidimensional indices, which include anemia, were calculated by adding three points to the previous values in cases of anemia. The prediction results were previously calculated by adding one to four points, and adding three points was the numerical value that obtained the best qualifying results. These modified versions were termed BODEx-A3 and ADO-A3.

Ethical considerations

The study adhered to the ethical principles outlined in the Declaration of Helsinki and received approval from the Clinical Research Ethics Committee of the Elche Health Department Hospital General (approval number: PI 31/2019). Written informed consent was obtained from all participants before their inclusion in the study.

Statistical analysis

The scores’ ability to predict one-year and three-year mortality was assessed by a receiver operating characteristic (ROC) analysis and the area under the ROC curve (AUC). The comparison of the AUC was performed using DeLong's test [14]. Sensitivity and specificity values were established at the best discriminative cutpoint identified by Youden's index. Statistical analysis was performed using the R software (R Foundation for Statistical Computing, Vienna, Austria: https://www.R-project.org/). A p＜0.05 denoted a statistically significant difference.

## Results

A total of 141 patients admitted to the hospital due to COPD exacerbation were included in the study, mostly males (82%). Two additional patients were excluded from the study due to the lack of required follow-up. Table 1 details the patients’ characteristics. About one-third of patients died during the three-year follow-up period. Of the 48 patients who died at three years, only three were women.

**Table 1:** Baseline characteristics of the patients Values are mean ± standard deviation unless otherwise indicated. n: number, mMRC: modified Medical Research Council scale, FEV1: forced expiratory volume in the first second, BMI: body mass index, BODEx: body mass index, airflow obstruction, dyspnea, and exacerbation, ADO: age, dyspnea severity, and airflow obstruction

Characteristics	Values
Number of patients	141
Age (years)	73 ± 10
Gender (female/male), n	26/115
Current smokers, n (%)	55 (39%)
Smoking (packs-year)	59 ± 27
Dyspnea (mMRC)	2.2 ± 0.9
No exacerbation in the previous year (n, %)	69 (49%)
Severe exacerbations (previous year) (n)	1.3 ± 1.7
FEV1 (% of reference)	46 ± 17
Anemia, n (% of patients)	34 (24%)
BODEx (range 0-9)	3.98 ± 2.10
BODEx-A3 (range 0-12)	4.70 ± 2.60
ADO (range 0-10)	5.26 ± 1.75
ADO-A3 (range 0-13)	5.98 ± 2.39
BMI (kg/m2)	27 ± 6
Mortality, 1 year (n, %)	21 (15%)
Mortality, 3 years (n, %)	48 (34%)

The prediction of one-year mortality was slightly higher with the new BODEx-A3 index compared to BODEx and with the new ADO-A3 index compared to ADO (Tables 2-3). The predictive capacity for three-year mortality was significantly higher with the BODEx-A3 and ADO-A3 indices compared to when anemia was not included in the variables (Tables 2-3).

**Table 2:** Prediction of mortality for BODEx and BODEx-A3 indices DeLong's test, p-values less than 0.05 were considered statistically significant. BODEx: body mass index, airflow obstruction, dyspnea, and exacerbation cut-off point 6, BODEX-A3: body mass index, airflow obstruction, dyspnea, and exacerbation cut-off point 7, established by Youden’s index, AUC: area under the curve

Predictive capacity		BODEx	BODEx-A3	p-value
1 year	AUC	0.78	0.83	0.17
	Sensitivity	62%	71%	
	Specificity	80%	87%	
3 years	AUC	0.67	0.77	<0.001
	Sensitivity	42%	50%	
	Specificity	82%	92%	

**Table 3:** Prediction of mortality for ADO and ADO-A3 indices DeLong's test, p-values less than 0.05 were considered statistically significant. ADO: age, dyspnea severity, and airflow obstruction cut-off point 6, ADO-A3: age, dyspnea severity, and airflow obstruction cut-off point 7, established by Youden’s index, AUC: area under the curve

Predictive capacity		ADO	ADO-A3	p-value
1 year	AUC	0.78	0.81	0.45
	Sensitivity	62%	81%	
	Specificity	83%	73%	
3 years	AUC	0.77	0.82	0.038
	Sensitivity	79%	69%	
	Specificity	65%	82%	

**Figure 1:**
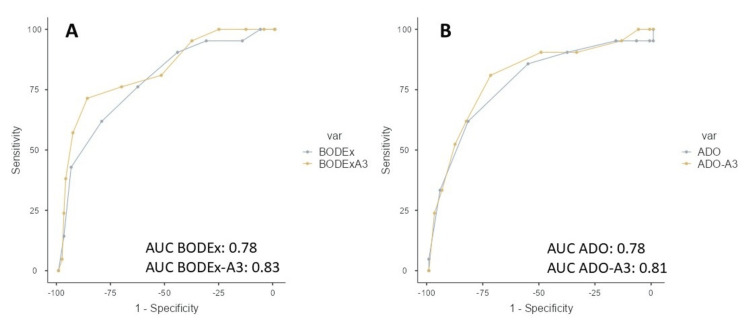
ROC curve and AUC of multidimensional indices for predicting one-year mortality A: BODEx and BODEx-A3, B: ADO and ADO-A3 ROC: receiver operating characteristic, AUC: area under the curve, BODEx: body mass index, airflow obstruction, dyspnea, and exacerbation, ADO: age, dyspnea severity, and airflow obstruction

**Figure 2:**
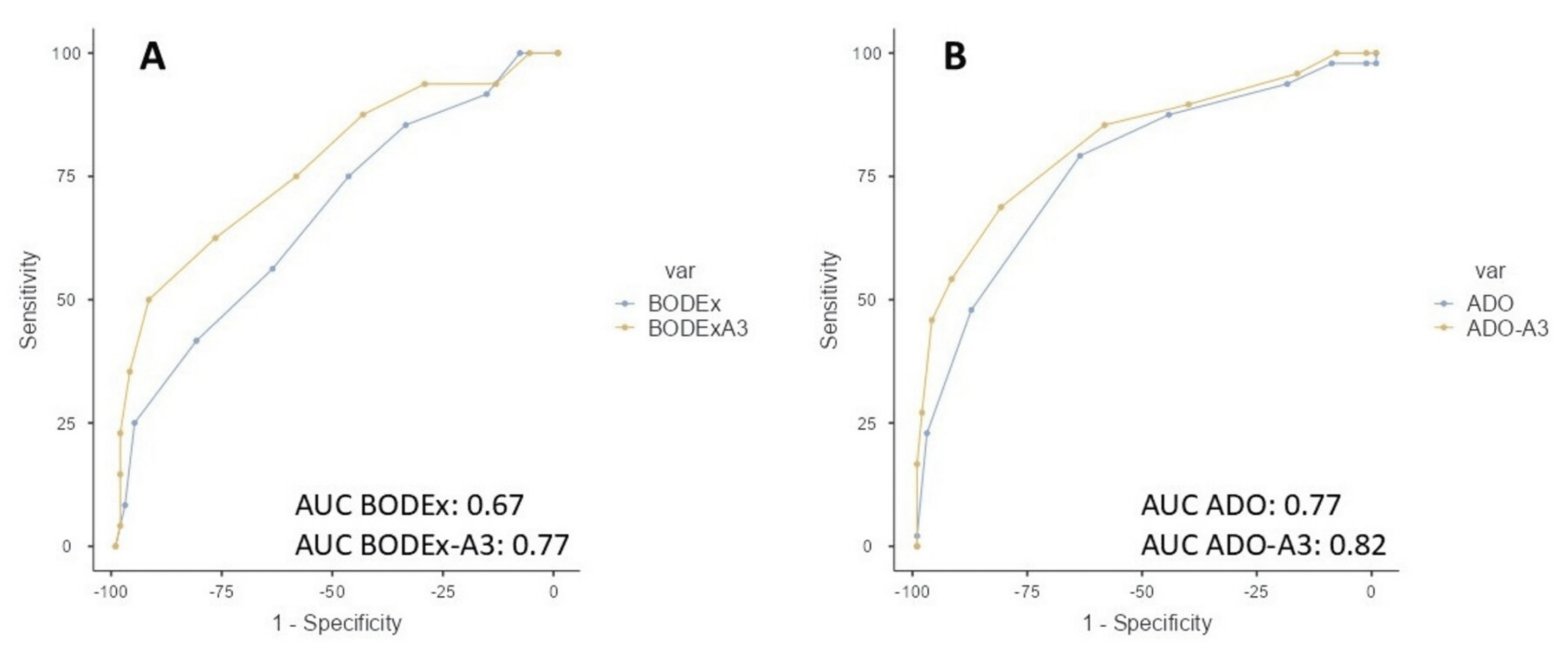
ROC curve and AUC of multidimensional indices for predicting three-year mortality A: BODEx and BODEx-A3, B: ADO and ADO-A3, values BODEx vs. BODEx-A3, p=0.001; ADO vs. ADO-A3, p=0.038 ROC: receiver operating characteristic, AUC: area under the curve, BODEx: body mass index, airflow obstruction, dyspnea, and exacerbation, ADO: age, dyspnea severity, and airflow obstruction

Figure 1 shows the AUCs for predicting survival at one year for the evaluated indices. Figure 2 shows the AUCs for predicting survival at three years.

## Discussion

This study shows that adding anemia to the best multidimensional indices predictors of COPD mortality improves its predictive ability at one and especially at three years. Adding three points to the usual indices when anemia is detected on admission is a simple adjustment that enhances the ability to predict mortality.

It has been clearly demonstrated that anemia is a strong predictor of mortality in patients admitted due to COPD exacerbation [12]. Anemia is frequently found in patients with COPD, but the exact mechanism through which it develops in these patients has not been definitively established. Most of the literature has focused on chronic disease-related anemia, particularly involving the hepcidin‐ferroportin axis and the cytokine-mediated development of erythropoietin resistance [15]. Despite its prevalence and clear association with mortality, anemia has not been included in the various indices designed to predict mortality in COPD patients.

Assessing the risk of near-term mortality provides crucial clinical information, particularly when evaluating the need for palliative care or determining eligibility for lung transplantation. Palliative care plays a key role in the comprehensive management of COPD patients, yet identifying those who would benefit from it remains challenging [16]. Research highlights the importance of integrating palliative care early, well before the end of life, to offer comprehensive support to both patients and their caregivers [17]. However, existing criteria for initiating palliative care based on poor prognosis in COPD patients over the short or medium term lack sufficient reliability. Our findings may help guide clinical decisions in this area.

Selecting COPD patients for lung transplantation is a complex and multifaceted decision. The BODE index has been used as the prognostic model of choice for selecting candidates for lung transplantation in COPD patients; however, several studies have concluded that the BODE index overestimates mortality [18]. Given that the inclusion of anemia in the BODEx index has significantly improved its ability to predict mortality, it would be reasonable to evaluate BODEx-A3 or ADO-A3 for this indication.

We report AUC values to evaluate the capacity to accurately classify patients based on their risk of death within specific time periods. Since the primary purpose of AUC values is often to compare the discriminative ability of different models [19], their assessment is particularly relevant to the objectives of this study. In general, AUC values above 0.7 are considered fair, and values above 0.8 are considered good or very good [19]. Previous studies have reported AUC values above 0.7 for both BODE and ADO in predicting one-year mortality in COPD patients [20]. In our cohort, both BODEx and ADO had an AUC of 0.78, and when anemia was included, both indices reached values above 0.8.

A network meta-analysis identified BODE plus exacerbation and ADO as the scores with the strongest ability to predict three-year mortality in COPD patients [21]. Our study demonstrates that the new indices, BODEx-A3 and ADO-A3, significantly enhance predictive accuracy.

Sensitivity and specificity values are presented using Youden's index as the selected cut-off to standardize data presentation using consistent criteria. However, for clinical decision-making, it may be preferable to present sensitivity and specificity values for different cut-off points. We did not provide these values because this study represents a first-approach analysis, which should be externally validated with data from other centers, ideally including a larger sample size and a higher proportion of female patients.

Due to the small number of female COPD-related deaths in this series, it was not possible to evaluate the usefulness of the new indices by gender. This is a limitation of the study, along with the fact that it was conducted at a single center, making replication in other settings necessary. However, a key strength of the study is that it was conducted in a real-world clinical setting with consecutive patients.

## Conclusions

We have defined novel multidimensional indices (BODEx-A3 and ADO-A3) for predicting short- and long-term mortality in patients hospitalized with COPD exacerbation. These new indices are obtained by adding three points to the value of the usual calculation of the BODEx and ADO indices in patients who present anemia on admission for COPD. We found that adding anemia status to the best existing predictor indices of COPD mortality significantly improves their predictive capacity. This represents a significant contribution to the therapeutic planning of patients requiring hospitalization for COPD exacerbation, including initiating palliative care, considering lung transplantation, or designing other specific interventions.
